# Conserved regions of the regulatory subunit Spo7 are required for Nem1–Spo7/Pah1 phosphatase cascade function in yeast lipid synthesis

**DOI:** 10.1016/j.jbc.2023.104683

**Published:** 2023-04-06

**Authors:** Ruta Jog, Gil-Soo Han, George M. Carman

**Affiliations:** Department of Food Science and the Rutgers Center for Lipid Research, New Jersey Institute for Food, Nutrition, and Health, Rutgers University, New Brunswick, New Jersey, USA

**Keywords:** phosphatidate, diacylglycerol, triacylglycerol, lipid synthesis, yeast, PA phosphatase, Nem1, Spo7, Pah1

## Abstract

In the yeast *Saccharomyces cerevisiae*, the Nem1–Spo7 complex is a protein phosphatase that activates Pah1 phosphatidate phosphatase at the nuclear–endoplasmic reticulum membrane for the synthesis of triacylglycerol. The Nem1–Spo7/Pah1 phosphatase cascade largely controls whether phosphatidate is partitioned into the storage lipid triacylglycerol or into membrane phospholipids. The regulated synthesis of the lipids is crucial for diverse physiological processes during cell growth. Spo7 in the protein phosphatase complex is required as a regulatory subunit for the Nem1 catalytic subunit to dephosphorylate Pah1. The regulatory subunit contains three conserved homology regions (CR1, CR2, and CR3). Previous work showed that the hydrophobicity of LLI (residues 54–56) within CR1 is important for Spo7 function in the Nem1–Spo7/Pah1 phosphatase cascade. In this work, by deletion and site-specific mutational analyses, we revealed that CR2 and CR3 are also required for Spo7 function. Mutations in any one of the conserved regions were sufficient to disrupt the function of the Nem1–Spo7 complex. We determined that the uncharged hydrophilicity of STN (residues 141–143) within CR2 was required for Nem1–Spo7 complex formation. In addition, the hydrophobicity of LL (residues 217 and 219) within CR3 was important for Spo7 stability, which indirectly affected complex formation. Finally, we showed the loss of Spo7 CR2 or CR3 function by the phenotypes (*e.g.*, reduced amounts of triacylglycerol and lipid droplets, temperature sensitivity) that are attributed to defects in membrane translocation and dephosphorylation of Pah1 by the Nem1–Spo7 complex. These findings advance knowledge of the Nem1–Spo7 complex and its role in lipid synthesis regulation.

In the yeast *Saccharomyces cerevisiae*, the Nem1–Spo7/Pah1 phosphatase cascade has emerged as one of the most important sequence of phosphatase reactions in lipid synthesis (1, 2, 3, 4, 5, 6). The enzyme cascade largely controls whether the key lipid intermediate phosphatidate (PA) is partitioned into membrane phospholipids for cell growth or into triacylglycerol (TAG) for lipid storage (7). Pah1 is an Mg^2+^-dependent PA phosphatase that catalyzes the dephosphorylation of PA to produce diacylglycerol (DAG) (Fig. 1*A*), which is then used for the synthesis of TAG (8, 9, 10). The PA phosphatase is more active as cells progress into the stationary phase when TAG accumulates at the expense of phospholipids (7, 11, 12). In contrast, the enzyme is less active in the exponential phase of growth (7, 12), and its substrate PA is mainly converted to the CDP-DAG that is used for the synthesis of phospholipids (2, 3). The loss of Pah1 function causes a plethora of physiological changes (reviewed by Kwiatek *et al.* (6)) that ultimately leads to a shortened chronological life span (13) with apoptotic cell death in the stationary phase (14). Some of the *pah1*Δ phenotypes associated with PA accumulation (*e.g.*, nuclear–endoplasmic reticulum [ER] membrane expansion) are governed by Dgk1 DAG kinase, the enzyme that converts DAG to PA (15, 16).

**Figure 1:**
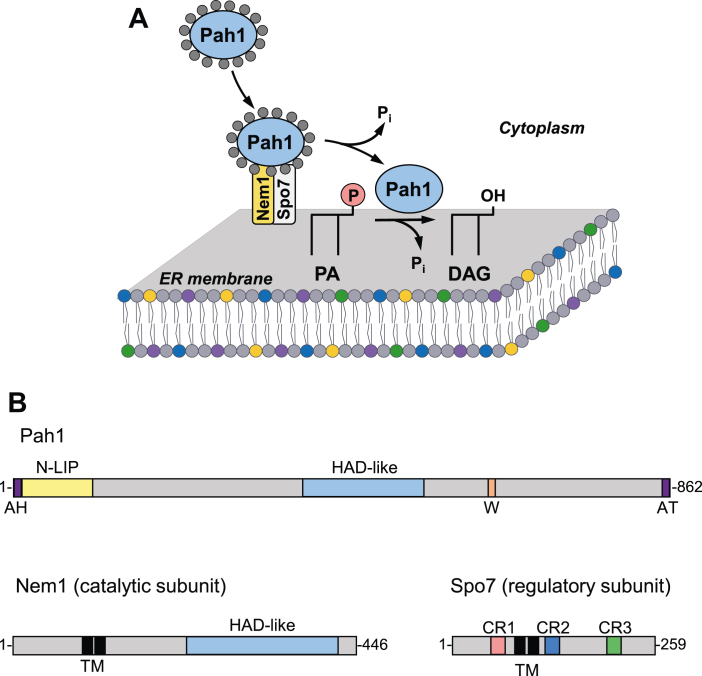
**Model for the Nem1–Spo7/Pah1 phosphatase cascade and domains/regions of Pah1, Nem1, and Spo7.***A*, phosphorylated Pah1 (*small grey circles*) translocates from the cytoplasm to the endoplasmic reticulum (ER) membrane through its recruitment and dephosphorylation by the Nem1–Spo7 protein phosphatase complex. Dephosphorylated Pah1 on the membrane catalyzes the dephosphorylation of PA to produce DAG. *B*, the diagram shows the domains/regions of Pah1 (*upper*), Nem1 (*lower left*), and Spo7 (*lower right*). Pah1 contains the N-terminal amphiphilic helix (AH) for membrane interaction (28), N-LIP and haloacid dehalogenase (HAD)-like domains that form the catalytic core (10, 42, 95), a conserved tryptophan (W) for Pah1 function (96), and C-terminal acidic tail (AT) for interaction with the Nem1–Spo7 complex (29). Nem1 contains the HAD-like catalytic domain and transmembrane (TM) region. Spo7 contains CR1 (*pink*), CR2 (*blue*), CR3 (*green*), and the TM region. DAG, diacylglycerol; PA, phosphatidate.

Pah1 function is mainly regulated by the post-translational modifications of phosphorylation and dephosphorylation (17) (Fig. 1*A*). In general, the enzyme phosphorylation is associated with the loss of function, whereas its dephosphorylation is associated with the gain of function (17). The enzyme phosphorylation, which is mediated by multiple protein kinases (*e.g.*, cyclin-dependent protein kinases Pho85-Pho80 (18) and Cdc28-cyclin B (19), glycogen synthase kinase homolog Rim11 (20), casein kinases I (21) and II (22), and protein kinases A (23) and C (24)), controls its cellular location, catalytic activity, and protein stability (17, 25, 26). Some of the kinase-specific sites of Pah1 phosphorylation are overlapping, and the enzyme phosphorylation by one protein kinase may affect its subsequent phosphorylation by another protein kinase (reviewed by Khondker *et al.* (17)). Perhaps the most important regulatory role of Pah1 phosphorylation, which occurs on the seven sites targeted by the Pho85–Pho80 protein kinase complex (18), is the sequestration of the enzyme in the cytoplasm to prevent it from accessing to the substrate PA in the nuclear–ER membrane (17, 18, 19, 27, 28, 29).

Nem1 (catalytic subunit)–Spo7 (regulatory subunit) complex (30) is essential to Pah1 function; it is responsible for the recruitment and dephosphorylation of Pah1 at the nuclear–ER membrane (17, 27, 28, 29, 31, 32) (Fig. 1). By nature of their enzyme–substrate relationship, the interaction of Pah1 with the Nem1–Spo7 complex is transient and difficult to observe (33). Following its dephosphorylation, Pah1 hops onto and scoots along the membrane to recognize PA for the production of DAG (34) (Fig. 1*A*). Coincidentally, the Pah1 substrate PA stimulates Nem1–Spo7 phosphatase activity (35). Interestingly, the protein phosphatase complex catalyzing Pah1 dephosphorylation (31, 32) is itself regulated by phosphorylation (36, 37, 38). For example, the Nem1 and Spo7 subunits are phosphorylated by protein kinases A and C (37, 38) with opposing effects on the function of the phosphatase complex in TAG synthesis. The Nem1–Spo7 phosphatase activity is stimulated by protein kinase C (38) but inhibited by protein kinase A (37). In addition, the prephosphorylation of Nem1–Spo7 by protein kinase C inhibits the protein kinase A phosphorylation of Nem1, whereas prephosphorylation of the complex by protein kinase A inhibits the protein kinase C phosphorylation of Spo7 (38). Yet another layer of complexity is the inhibition of the Nem1–Spo7 complex by the ER-associated protein Ice2 (39). The mechanism by which Ice2 inhibits the Nem1–Spo7 dephosphorylation of Pah1 has yet to be defined (39).

The interaction of Spo7 with Nem1 is required for Nem1 activity (30) and stability (36, 39, 40). The regulatory subunit is conserved in eukaryotes with three homology regions (CR1, CR2, and CR3) (41) (Fig. 1*B*). CR1 is located at the N-terminal region, whereas CR2 and CR3 are located in the middle and at the C-terminal region, respectively. The hydrophobic amino acids LLI (residues 54–56) within CR1 are required for Spo7 interaction with Nem1 and thus for the function of the phosphatase complex in TAG synthesis (40). In the present work, we sought to determine the structural requirements of CR2 and CR3 for Spo7 function. Deletion and site-specific mutational analyses of Spo7 revealed that its CR2 and CR3 are also required for Spo7 function. The uncharged hydrophilicity of STN (residues 141–143) within CR2 was required for Nem1–Spo7 complex formation. The hydrophobicity of LL (residues 217 and 219) within CR3 was important for Spo7 stability, which indirectly affected complex formation. The loss of Spo7 CR2 or CR3 function was shown by the phenotypes (*e.g.*, reduced amounts of TAG and lipid droplets, temperature sensitivity) that are attributed to the defects of Pah1 in its membrane translocation and dephosphorylation by the Nem1–Spo7 complex. These findings advance the understanding of the Nem1–Spo7 complex in the regulation of lipid synthesis.

## Results

### Spo7 conserved regions are required for Nem1–Spo7/Pah1 function in TAG synthesis

**Figure 2:**
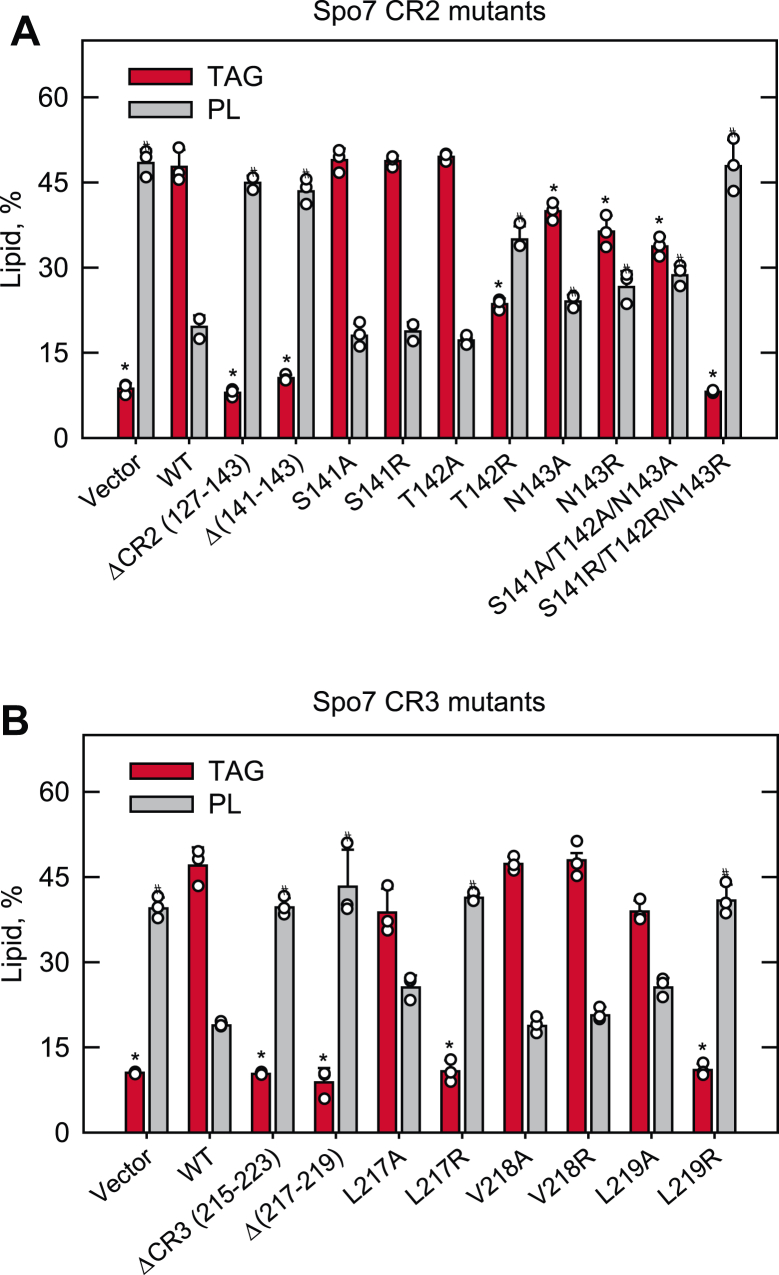
**TAG and phospholipid synthesis of cells expressing Nem1 and Spo7 with CR2 and CR3 mutations.** The *spo7*Δ mutant (GHY67) was transformed with pGH443 or its derivative for the expression of the CR2 (*A*) or CR3 (*B*) mutant allele of *SPO7*. The transformants were grown at 30 °C to the stationary phase in SC-Leu medium containing [2-^14^C]acetate (1 μCi/ml). Lipids were extracted from the radiolabeled cells, separated by TLC, subjected to phosphorimaging, and quantified by ImageQuant analysis. The levels of TAG and phospholipids (PLs) were normalized to total chloroform-soluble lipids. The data are means ± SD (error bars) from biological triplicates. The individual data points are also shown. ∗*p* < 0.05 *versus* TAG of WT cells. #*p* < 0.05 *versus* phospholipid of WT cells. SC-Leu, synthetic dropout media without leucine; TAG, triacylglycerol.

To examine the importance of Spo7 CR2 (residues 127–143) and CR3 (residues 215–223) in lipid synthesis, we generated the *SPO7* alleles lacking the conserved regions (Table 1) and expressed them in the *spo7*Δ mutant. The mutant alleles were constructed on a single copy plasmid driven by the native *SPO7* promoter to approximate the endogenous gene expression level. We assessed the function of the *SPO7* alleles expressed in *spo7*Δ cells by analyzing the level of TAG, which is controlled by the Nem1–Spo7/Pah1 phosphatase cascade. Lipids were extracted and analyzed from [2-^14^C]acetate-labeled cells in the stationary phase when the TAG level is highest (7, 10, 14). As described previously (40), the *spo7*Δ cells (*i.e.*, vector control) showed a five fold lower level of TAG when compared with those expressing WT Spo7 (Fig. 2). The defect of the mutant cells in TAG synthesis was also reflected by a 2.5-fold increase in the level of phospholipids. The altered lipid levels are attributed to the lack of Pah1 function in producing DAG for TAG synthesis and the concomitant accumulation of PA, which is converted to phospholipids *via* CDP-DAG and derepresses the expression of the UAS_INO_-containing phospholipid biosynthetic genes (6, 7, 10, 32, 42, 43, 44). Unlike WT *SPO7*, the expression of the mutant alleles lacking CR2 (Fig. 2*A*) or CR3 (Fig. 2*B*) did not restore the altered levels of TAG and phospholipids, indicating that the conserved regions are essential for the protein function. Further mutational analyses showed that the loss-of-function effects of the CR2 and CR3 deletions were mimicked by the deletions of the STN (residues 141–143) and LVL (residues 217–219) sequences, respectively, within the conserved regions. These amino acids were changed individually and in combination to hydrophobic (*e.g.*, alanine) or hydrophilic (*e.g.*, arginine) amino acids and examined for their mutational effects.

**Table 1:** Plasmids used in this study

Plasmid	Relevant characteristics	Source or reference
YCplac111	Single-copy number *E. coli*/yeast shuttle vector with *LEU2*	(97)
Derivative		
YCplac111-*GAL1/10*-*NEM1*-PtA	*NEM1*-PtA under control of *GAL1/10* promoter inserted into YCplac111	(30)
pRS415	Single-copy number *E. coli*/yeast shuttle vector with *LEU2*	(80)
Derivatives		
pGH443	*SPO7* with its own promoter inserted into pRS415	(37)
pGH443-ΔCR1 (52–64)	*SPO7* lacking CR1 residues 52–64	This study
pGH443-ΔCR2 (127–143)	*SPO7* lacking CR2 residues 127–143	This study
pGH443-Δ (132–134)	*SPO7* lacking residues 132–134	This study
pGH443-Δ (141–143)	*SPO7* lacking residues 141–143	This study
pGH443-ΔCR3 (215–223)	*SPO7* lacking CR3 residues 215–223	This study
pGH443-Δ (217–219)	*SPO7* lacking residues 217–219	This study
pGH443-R130A	*SPO7* with the R130A mutation	This study
pGH443-P135A	*SPO7* with the P135A mutation	This study
pGH443-S141A	*SPO7* with the S141A mutation	This study
pGH443-S141R	*SPO7* with the S141R mutation	This study
pGH443-T142A	*SPO7* with the T142A mutation	This study
pGH443-T142R	*SPO7* with the T142R mutation	This study
pGH443-N143A	*SPO7* with the N143A mutation	This study
pGH443-N143R	*SPO7* with the N143R mutation	This study
pGH443-K216A	*SPO7* with the K216A mutation	This study
pGH443-L217A	*SPO7* with the L217A mutation	This study
pGH443-L217R	*SPO7* with the L217R mutation	This study
pGH443-V218A	*SPO7* with the V218A mutation	This study
pGH443-V218R	*SPO7* with the V218R mutation	This study
pGH443-L219A	*SPO7* with the L219A mutation	This study
pGH443-L219R	*SPO7* with the L219R mutation	This study
pGH443-P221A	*SPO7* with the P221A mutation	This study
pGH443-R222A	*SPO7* with the R222A mutation	This study
pGH443-R130A/R136A/R137A	*SPO7* with the R130A, R136A, and R137A mutations	This study
pGH443-S141A/T142A/N143A	*SPO7* with the S141A, T142A, and N143A mutations	This study
pGH443-S141R/T142R/N143R	*SPO7* with the S141R, T142R, and N143R mutations	This study
pRS314	Single-copy number *E. coli*/yeast shuttle vector with *TRP1*	(80)
Derivatives		
pRS314-*GAL1/10-SPO7*	*SPO7* under the control of *GAL1/10* promoter inserted in pRS314	(37)
pRS314-*GAL1/10-SPO7*-ΔCR1 (52–64)	*SPO7* lacking CR1 residues 52–64	This study
pRS314-*GAL1/10-SPO7*-ΔCR2 (127–143)	*SPO7* lacking CR2 residues 127–143	This study
pRS314-*GAL1/10-SPO7*-ΔCR3 (215–223)	*SPO7* lacking CR3 residues 215–223	This study
pRS314-*GAL1/10-SPO7*- S141A/T142A/N143A	*SPO7* with S141A, T142A, and N143A mutations	This study
pRS314-*GAL1/10-SPO7*-S141R/T142R/N143R	*SPO7* with S141R, T142R, and N143R mutations	This study
pRS314-*GAL1/10-SPO7*-L217A	*SPO7* with the L217A mutation	This study
pRS314-*GAL1/10-SPO7*-L217R	*SPO7* with the L217R mutation	This study
pRS314-*GAL1/10-SPO7*-L219A	*SPO7* with the L219A mutation	This study
pRS314-*GAL1/10-SPO7*-L219R	*SPO7* with the L219R mutation	This study
pGH452	*PAH1*-PtA under the control of *GAL1/10* promoter in pYES2	(81)

The T142R, N143A, and N143R mutations caused 1.2- to 2-fold decreases in TAG levels and 1.2- to 1.8-fold increases in phospholipid levels when compared with the WT control (Fig. 2*A*). The S141A, S141R, and T142A mutations, however, did not show significant mutational effects. The triple alanine mutations (S141A/T142A/N143A) did not enhance the mutational effects of N143A, but the triple arginine mutations (S141R/T142R/N143R) caused the same effects on lipid composition displayed by the Δ(141–143) mutations (Fig. 2*A*). These effects could be attributed to the T142R and N143R mutations.

The alanine mutations for the LVL sequence did not impart significant mutational effects on the lipid levels (Fig. 2*B*). However, the L217R and L219R mutations caused changes in the amounts of TAG (4.2- to 4.7-fold decrease) and phospholipids (2.1- to 2.3-fold increase) that were similar to those caused by the Δ(217–219) mutations. The V218R mutation, however, did not cause alterations in lipid composition. Thus, the hydrophobicity of Leu-217 and Leu-219 within CR3 is important for Spo7 function.

### Spo7 conserved regions are required for lipid droplet formation

**Figure 3:**
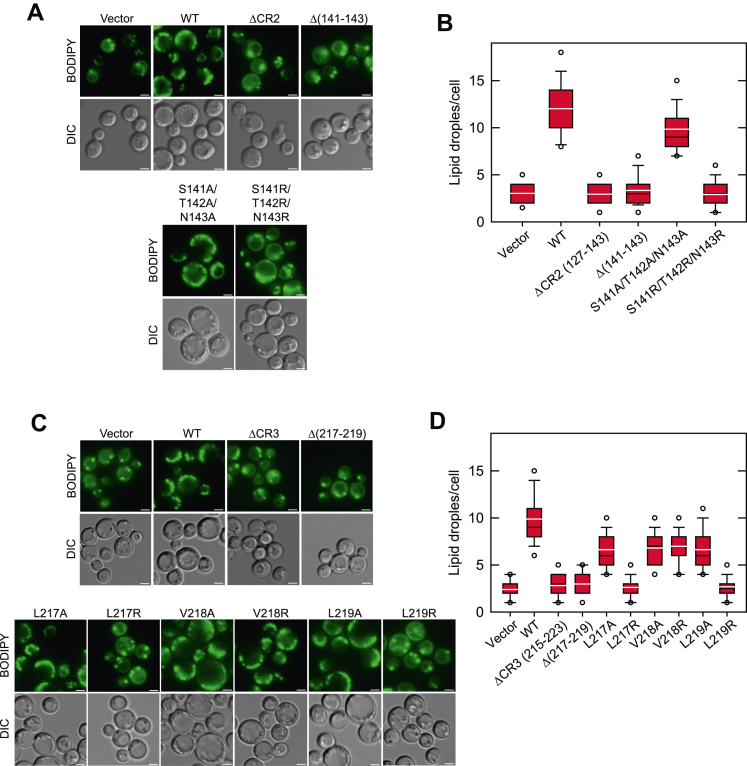
**Lipid droplet formation of cells expressing Nem1 and Spo7 with CR2 and CR3 mutations.** The *spo7*Δ mutant (GHY67) was transformed with pGH443 or its derivative for the expression of the CR2 (*A*) or CR3 (*C*) mutant allele of *SPO7*. The cells were grown at 30 °C in SC-Leu medium to the stationary phase and then stained with BODIPY 493/503. The stained lipid droplets were visualized by fluorescence microscopy, and the number of lipid droplets was counted from ≥200 cells (≥4 fields of view). *A* and *C*, the images shown are representative of multiple fields of view. *White bar* represents 1 μm. *B* and *D*, the data are presented by the box plot. The *black* and *white lines* are the median and mean values, respectively, and the *white circles* are the outlier data points of the 5th and 95th percentile. DIC, differential interference contrast; SC-Leu, synthetic dropout media without leucine.

The Nem1–Spo7/Pah1 phosphatase cascade produces the DAG that is acylated to TAG at the nuclear–ER membrane (6, 10). TAG is then packaged and stored in lipid droplets that are primarily localized to the cytoplasm (45). Loss of Nem1–Spo7/Pah1 function, as caused by the *spo7*Δ mutation, results in a significant reduction in lipid droplet formation (40). Accordingly, we examined the effects of the Spo7 CR2 and CR3 mutations on the abundance of cytoplasmic lipid droplets in stationary phase cells (Fig. 3). As described previously (40), the *spo7*Δ cells (*i.e.*, vector control) showed a threefold lower number of lipid droplets when compared with those expressing WT *SPO7* (Fig. 3). Consistent with their effects on the TAG content, the ΔCR2, Δ(141–143), and S141R/T142R/N143R (Fig. 3, *A* and *B*) and the ΔCR3, Δ(217–219), L217R, and L219R (Fig. 3, *C* and *D*) mutations caused the reduction of lipid droplet formation that is similar to that exhibited by the lack of *SPO7*. These observations provide additional support for the conclusion that CR2 and CR3 are required for Spo7 function.

### Spo7 conserved regions are required for cell growth at elevated temperature

**Figure 4:**
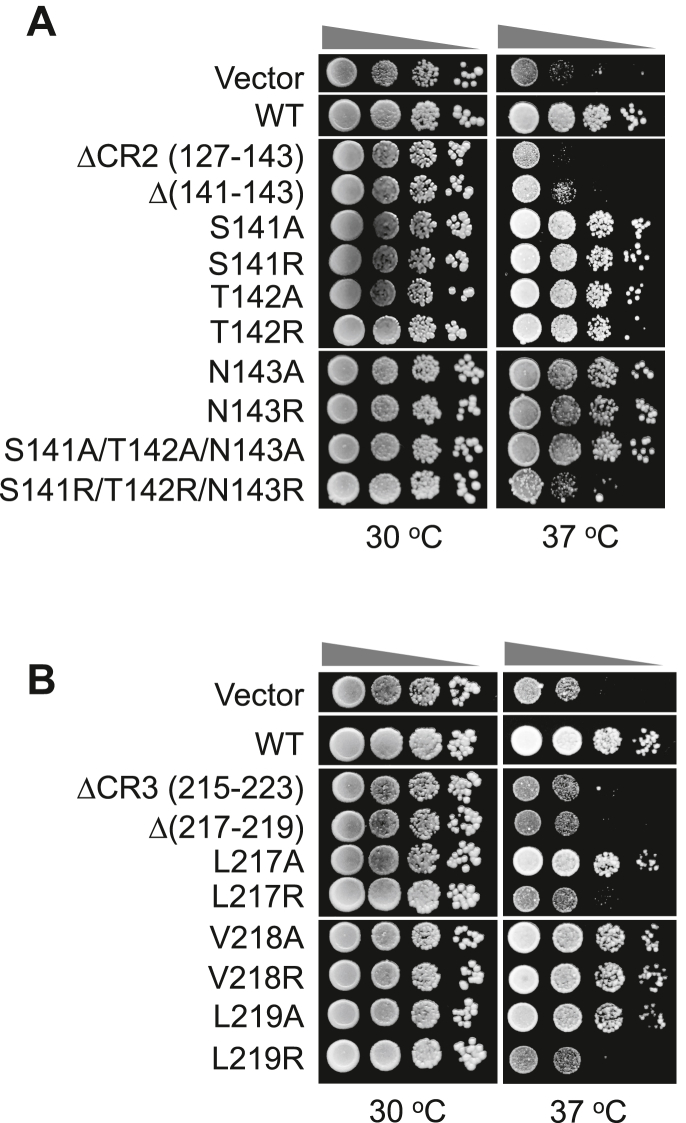
**Temperature sensitivity of cells expressing Nem1 and Spo7 with CR2 and CR3 mutations.** The *spo7*Δ mutant (GHY67) was transformed with pGH443 or its derivative for expression of the CR2 (*A*) or CR3 (*B*) mutant alleles of *SPO7*. The transformants were grown to saturation at 30 °C in SC-Leu medium. The cultures were adjusted to absorbance of 0.7 at 600 nm, serially diluted (10-fold) in SC-Leu medium, and spotted (5 μl) onto YPD plates. The growth of the transformant cells at 30 and 37 °C was scored after 3 days of incubation. The data are representative of three replicate experiments. SC-Leu, synthetic dropout media without leucine; YPD, yeast extract–peptone–dextrose.

The defect in the Nem1–Spo7/Pah1 phosphatase cascade results in a plethora of phenotypes, which include the aberrant expansion of the nuclear–ER membrane (30, 32), susceptibility to fatty acid–induced toxicity (14), hypersensitivity to oxidative stress (14), defects in autophagy (46, 47), cell wall integrity (48, 49), vacuole fusion and acidification (50, 51), and cell growth on nonfermentable carbon sources (10, 52), and at elevated temperatures (10, 32, 52). One of the most striking phenotypes because of the loss of Nem1–Spo7/Pah1 function is characterized by the inability of the mutant to grow at the elevated temperature of 37 °C. Accordingly, the importance of CR2 and CR3 for Spo7 function was scored by this phenotype. Whereas WT *SPO7* complements the temperature-sensitive phenotype of *spo7*Δ cells, the ΔCR2, Δ(141–143), and S141R/T142R/N143R (Fig. 4*A*) and ΔCR3, Δ(217–219), L217R, and L219R (Fig. 4*B*) alleles failed to complement the mutant phenotype.

### Spo7 conserved regions are required for the Nem1–Spo7-mediated membrane translocation of Pah1

**Figure 5:**
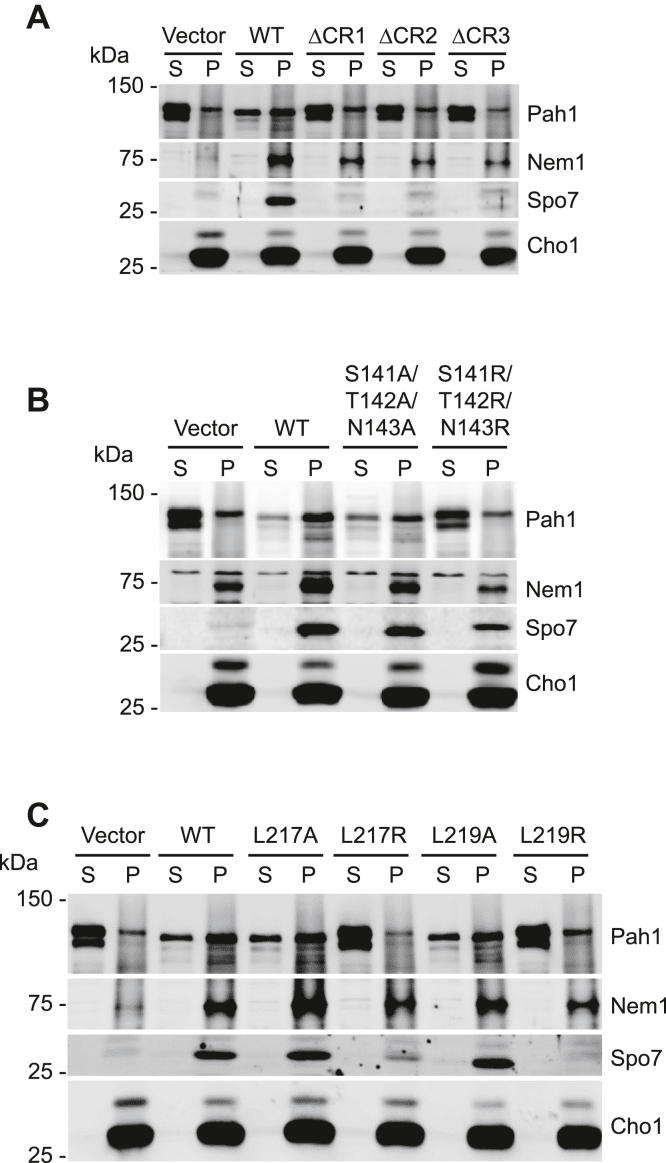
***In vitro* translocation of Pah1 to the membrane containing Nem1 and Spo7 with CR mutations.** Purified phosphorylated Pah1 (20 ng) was incubated for 20 min at 30 °C with the membranes (30 μg) prepared from *nem1*Δ *spo7*Δ *pah1*Δ (GHY85) cells coexpressing the plasmids YCplac111-*GAL1/10*-*NEM1*-PtA and pRS314-*GAL1/10-SPO7* (WT or ΔCR1, ΔCR2, ΔCR3 (*A*); CR2 (*B*); CR3 (*C*) mutant forms) in a total volume of 20 μl. Following the incubation, the reaction mixtures were fractionated by centrifugation at 100,000*g* for 1 h at 4 °C. The membrane pellet (P) was resuspended in the same volume of the supernatant (S), and equal volumes of the fractions were resolved by SDS-PAGE (10% polyacrylamide gel) and transferred to a polyvinylidene difluoride membrane. Membranes were probed with antibodies against Pah1, Nem1, Spo7, and Cho1 (ER membrane marker). Anti-Nem1 antibody raised against the residues 65 to 83 was used in *A* and *C*, and anti-Nem1 antibody raised against the residues 127 to 141 was used in *B*. Anti-Spo7 antibody raised against the residues 58 to 69 was used in *B*, and anti-Spo7 antibody raised against the residues 242 to 259 was used in *A* and *C*. The sequences used to raise the anti-Spo7 antibodies did not overlap with the regions of the Spo7 mutations. The positions of Pah1, Nem1, Spo7, and Cho1, and molecular mass standards are indicated. The band above Nem1 in *B* is a nonspecific signal because of a difference in the antibodies. The weak signal above Cho1 indicates the phosphorylated form of the protein by protein kinase A (75). The data shown are representative of four replicate experiments. ER, endoplasmic reticulum.

The Nem1–Spo7 complex is responsible for the recruitment of Pah1 to the nuclear–ER membrane (18, 19, 31, 32). To determine whether the conserved region mutations of Spo7 affect the translocation of Pah1 to the membrane, we examined its membrane association in an *in vitro* translocation assay. In this assay, purified phosphorylated Pah1 was incubated with the Pah1-free membrane containing Nem1 and Spo7 and then fractionated for detection of its membrane association (Fig. 5). In controls, most Pah1 remained in the soluble fraction when incubated with the Spo7-deficient membrane. However, the level of Pah1 was greatly reduced in the soluble fraction and showed a concomitant increase in the membrane fraction when incubated with the membrane containing WT Spo7, indicating that Pah1 was translocated to the membrane by the functional Nem1–Spo7 complex. The level of Pah1 translocated to the membrane fraction was detected at a reduced level because of its proteolytic degradation (25). The membrane translocation of Pah1 was reduced when incubated with the membranes prepared from the cells expressing the ΔCR1, ΔCR2, and ΔCR3 mutant forms of Spo7 (Fig. 5*A*). The arginine (S141R/T142R/N143R and L217R and L219R) but not the alanine (S141A/T142A/N143A and L217A and L219A) mutations of CR2 (Fig. 5*B*) and CR3 (Fig. 5*C*) similarly caused a reduction in the translocation of Pah1 to the membrane.

The relative abundance of the Spo7 conserved region mutant proteins that caused defects in Nem1–Spo7/Pah1 function (*e.g.*, lipid synthesis, lipid droplet formation, and the temperature sensitivity) was reduced when compared with the WT control or the point mutants that did not affect Spo7 function. This observation is not expected to be due to a defect in the expression of the proteins since all genetic constructs were expressed from the same plasmid. Instead, the mutations might affect Spo7 structure, causing reduced stability of the protein. As described previously (36, 39, 40), the reduced amount of Spo7 correlated with a reduction in the relative amount of Nem1 (Fig. 5).

### Spo7 conserved regions are essential for the Nem1–Spo7 activity on Pah1

**Figure 6:**
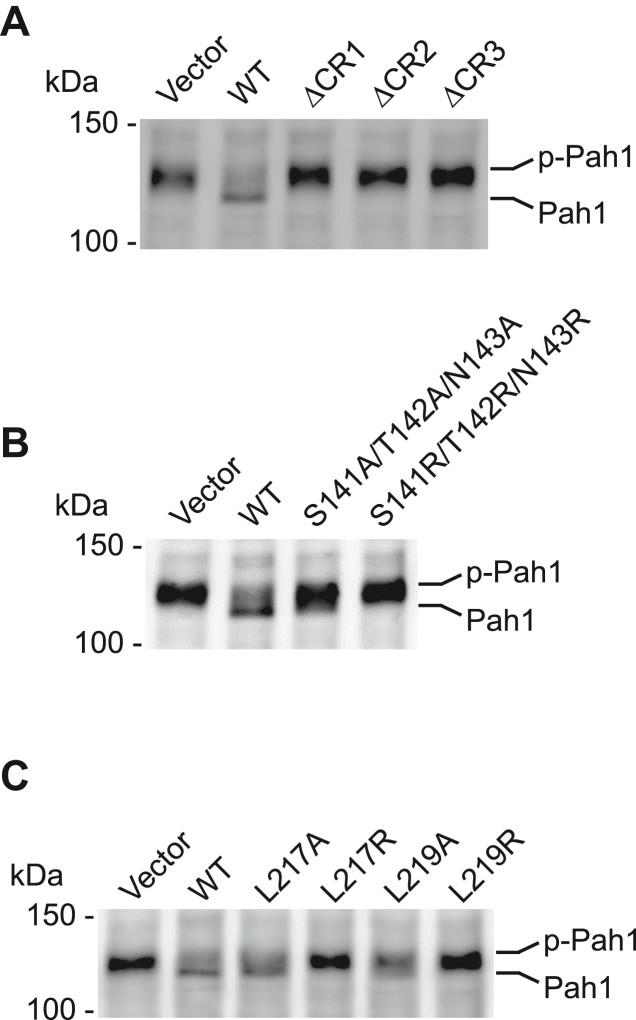
**Pah1 dephosphorylation by the membrane containing Nem1 and Spo7 with CR mutations.** Purified phosphorylated Pah1 (2.5 ng) was incubated for 20 min at 30 °C with the membranes (20 μg) prepared from *nem1*Δ *spo7*Δ *pah1*Δ (GHY85) cells coexpressing plasmids YCplac111-*GAL1/10*-*NEM1*-PtA and pRS314-*GAL1/10-SPO7* (WT or ΔCR1, ΔCR2, ΔCR3 (*A*); CR2 (*B*); CR3 (*C*) mutant forms) under the assay conditions for Nem1–Spo7 phosphatase activity (31). Following the incubation, the reaction mixtures resolved by SDS-PAGE (6% polyacrylamide gel), transferred to polyvinylidene difluoride membrane, and probed with anti-Pah1 antibody. The positions of Pah1 in the phosphorylated (*p-Pah1*) and dephosphorylated (*Pah1*) states and molecular mass standards are indicated. The data shown are representative of four independent experiments.

The dephosphorylation of Pah1 by the Nem1–Spo7 complex is shown by an increase in the electrophoretic mobility of the protein in SDS-PAGE (35, 40). Using purified phosphorylated Pah1, we examined its electrophoretic mobility upon incubation with the Pah1-free membranes containing Nem1 and Spo7. Pah1 incubated with the membrane containing WT Nem1-–Spo7 showed an increase in the electrophoretic mobility when compared with the vector control, indicating that the protein was converted to its dephosphorylated form (31, 35, 40) (Fig. 6). The dephosphorylation of Pah1 renders the protein unstable and prone to proteolytic degradation (25, 26). The abundance of Pah1 that was incubated with the WT Nem1–Spo7 complex–containing membranes was reduced when compared with the vector control (Fig. 6). As expected, incubation with the membranes lacking the Nem1–Spo7 complex (vector control) had no effect on the abundance of Pah1. Changes in the electrophoretic mobility of Pah1 and its abundance were not observed when the purified phosphorylated Pah1 was incubated with the membranes containing Spo7 with the ΔCR1, ΔCR2, and ΔCR3 mutations (Fig. 6*A*) and the arginine mutations of Ser-141/Thr-142/Asn-143 (Fig. 6*B*) and Leu-217 and Leu-219 (Fig. 6*C*) when compared with the vector control. Thus, these Spo7 mutations prevented the dephosphorylation of Pah1. However, the alanine mutations of the indicated CR2 and CR3 residues of Spo7 did not compromise the Nem1–Spo7 complex–mediated dephosphorylation of Pah1 (Fig. 6).

### Spo7 conserved regions mediate its complex formation with Nem1

**Figure 7:**
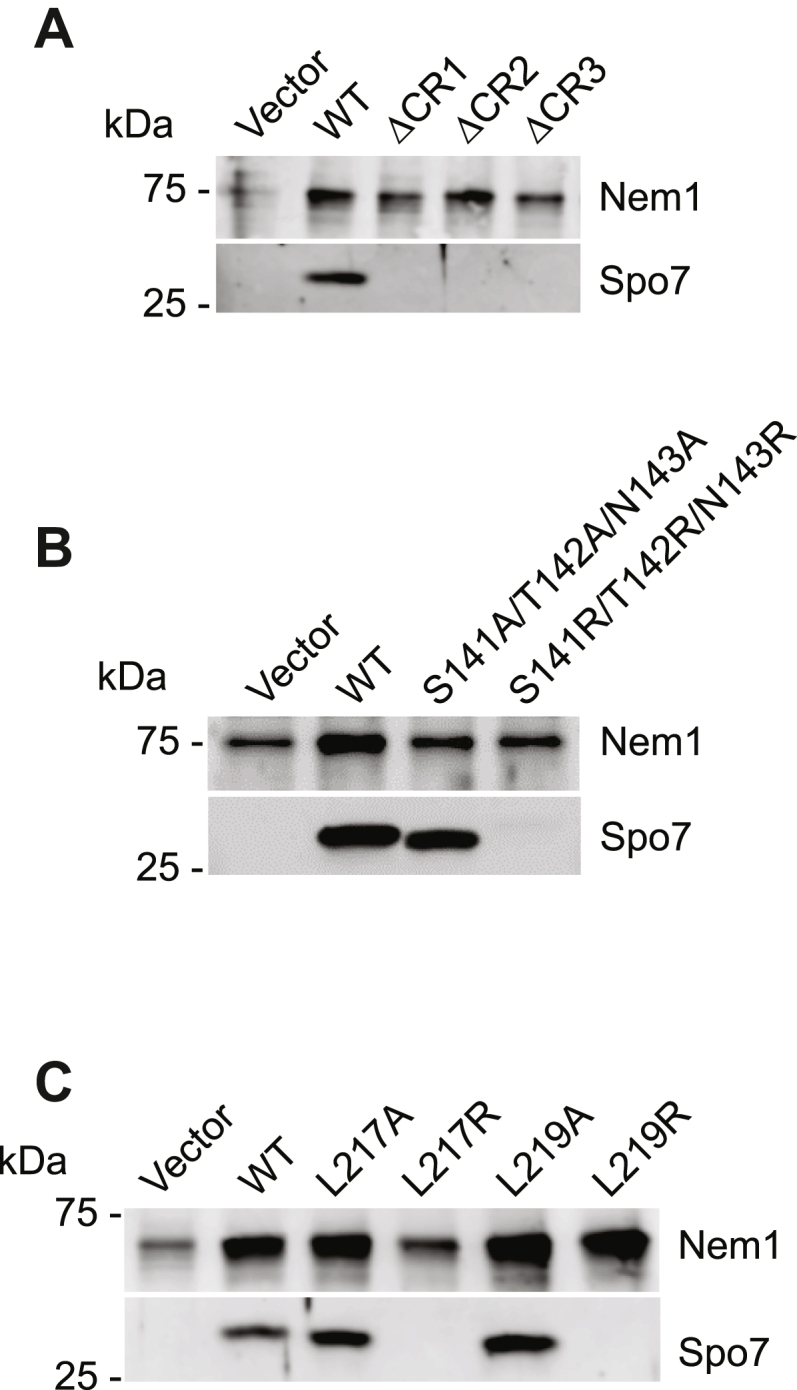
**Complex formation of Nem1 and Spo7 with CR mutations.** Protein A-tagged Nem1 was purified from the cell extracts of *nem1*Δ *spo7*Δ *pah1*Δ (GHY85) cells coexpressing plasmids YCplac111-*GAL1/10*-*NEM1*-PtA and pRS314-*GAL1/10-SPO7* (WT or ΔCR1, ΔCR2, ΔCR3 (*A*); CR2 (*B*); CR3 (*C*) mutant forms) by IgG-Sepharose affinity chromatography (30, 31). The affinity-purified Nem1 preparations were resolved by SDS-PAGE (12.5% polyacrylamide gel) and transferred to a polyvinylidene difluoride membrane. The membrane was probed with antibodies against Nem1 and Spo7. Anti-Nem1 antibody raised against the residues 65 to 83 was used in *A* and *C*, and anti-Nem1 antibody raised against the residues 127 to 141 was used in *B*. Anti-Spo7 antibody raised against the residues 58 to 69 was used in *B*, and anti-Spo7 antibody raised against the residues 242 to 259 was used in *A* and *C*. The sequences used to raise the anti-Spo7 antibodies did not overlap with the regions of the Spo7 mutations. The formation of the Nem1–Spo7 complex was scored by the presence of Spo7 in the affinity-purified Nem1 preparation (30). The positions of Nem1, Spo7, and molecular mass standards are indicated. The data shown are representative of three replicate experiments.

The catalytic function of Nem1 to dephosphorylate Pah1 is dependent on its interaction with Spo7 (30, 31, 32, 36, 41). Accordingly, we examined whether the CR2 and CR3 mutations of Spo7 affects its complex formation with Nem1 *in vivo*. In this analysis, protein A-tagged Nem1 was coexpressed with the WT and mutant forms of Spo7 followed by the isolation of the complex from cell extracts by affinity chromatography with IgG-Sepharose. Column effluents were examined for the presence of the complex by immunoblot analysis using anti-Nem1 and anti-Spo7 antibodies. The ΔCR2 and ΔCR3 mutations in Spo7 obviated the formation of the complex; the Spo7 protein was not detected in the effluent of the IgG-Sepharose affinity resin (Fig. 7*A*). The Leu-54 Leu-55 Ile-56 sequence within CR1 is required for Spo7 interaction with Nem1 (40), and as expected, the ΔCR1 mutation reduced the complex formation (Fig. 7*A*). The analysis was performed with the site-specific CR2 and CR3 mutants of Spo7 that had the greatest negative effects on the Spo7 physiological function. The S141R/T142R/N143R (Fig. 7*B*) and the L217R and L219R (Fig. 7*C*) mutations prevented the formation of the Nem1–Spo7 complex. The alanine mutations of these residues did not prevent the formation of the complex.

## Discussion

The protein phosphatase and lipid phosphatase reactions catalyzed by the Nem1–Spo7/Pah1 axis control the PA–DAG balance in yeast (2, 3, 6). By controlling the levels of PA and DAG (10, 14, 44), the phosphatase cascade influences the expression of several membrane lipid synthesis genes (32, 43) *via* the Opi1/Ino2–Ino4 Henry regulatory circuit (6, 53, 54, 55), the growth of the nuclear–ER membrane (30, 32), and the synthesis of TAG (10) and lipid droplets (45). The cascade also impacts on diverse physiological functions that include cell wall integrity (48, 49), vacuole homeostasis (50), target of rapamycin complex 1–mediated induction of autophagy (46), susceptibility to fatty acid–induced lipotoxicity (14), sensitivity to cold (56), heat (10, 15, 32, 52), and to oxidative stress (13), and growth on nonfermentable carbon sources (10, 42). The major mechanism by which the PA–DAG balance is regulated through multiprotein kinase phosphorylation of Pah1, which sequesters the enzyme in the cytoplasm, and the Nem1–Spo7 complex–mediated recruitment and dephosphorylation of Pah1, which permits the enzyme to associate with and dephosphorylate membrane-associated PA to produce DAG (17, 35).

The Nem1–Spo7/Pah1 phosphatase cascade is not unique to yeast. The homologous phosphatase cascade in mammalian cells is known as CTDNEP1-NEP1-R1/lipin 1 (41, 57, 58, 59, 60). Conservation of CTDNEP1-NEP1-R1 function is indicated by its complementation of the *nem1*Δ *spo7*Δ mutant phenotypes such as nuclear–ER membrane expansion and reductions in TAG and lipid droplet formation (41). Similar to yeast Pah1, mouse lipin 1 is a PA phosphatase (61) whose state of phosphorylation governs its subcellular localization (62, 63, 64, 65, 66), and when expressed in human cells, the CTDNEP1-NEP1-R1 dephosphorylates lipin 1 (41). The critical roles that the phosphatase cascade plays in humans and mice are typified by assorted lipinopathies (*e.g.*, lipodystrophy, insulin resistance, peripheral neuropathy, rhabdomyolysis) that result by loss of lipin 1 PA phosphatase function (57, 67, 68, 69, 70, 71).

Spo7 may be considered a key regulator in the Nem1–Spo7/Pah1 phosphatase cascade. Its complex formation with Nem1 plays a role in recruiting Pah1 to the phosphatase complex (29), and the regulatory subunit is essential for the catalytic function of Nem1 to dephosphorylate Pah1 (29, 30, 40). Moreover, the interaction serves to stabilize Nem1 (36, 39, 40). The hydrophobicity imparted by the LLI sequence (residues 54–56) within CR1 is important for Spo7 interaction with Nem1 (40). Here, we sought information on the roles of CR2 and CR3 to Spo7 function. Through deletion analysis, the sequences contained within CR2 and CR3 were shown to be important for Spo7 function as reflected in phenotypes (*e.g.*, reduced amounts of TAG and lipid droplets, temperature sensitivity) characteristic of a defect in Pah1 PA phosphatase activity. These mutant phenotypes can be attributed to reductions in the translocation of Pah1 to membranes and the Nem1–Spo7 complex–mediated dephosphorylation of Pah1; the mechanistic basis for these defects can be attributed to the reduction of Nem1–Spo7 complex formation.

The site-specific mutational analyses indicated that the combination of the uncharged hydrophilic residues Ser-141, Thr-142, and Asn-143 within CR2 was important for the formation of the Nem1–Spo7 complex. When these residues were changed to charged hydrophilic arginine residues (*e.g.*, S141R/T142R/N143R), the interaction of Spo7 with Nem1 was disrupted. However, when hydrophobic alanine was substituted for Ser-141, Thr-142, and Asn-143, the complex formation was not affected. The hydrophobic residues Leu-217 and Leu-219 within CR3 were important for Spo7 stability. The L217R and L219R mutations, which alter hydrophobicity, caused a reduction in Spo7 abundance. These mutations themselves may decrease Spo7 interaction with Nem1, resulting in their instability; or the mutations themselves may make Spo7 unstable, and thus unable to form a complex with Nem1. In either case, the complex formation was compromised by the mutations. Spo7 abundance was not affected by the L217A and L219A mutations that retain hydrophobicity at those sites in the protein and Nem1–Spo7 complex formation was not compromised.

**Figure 8:**
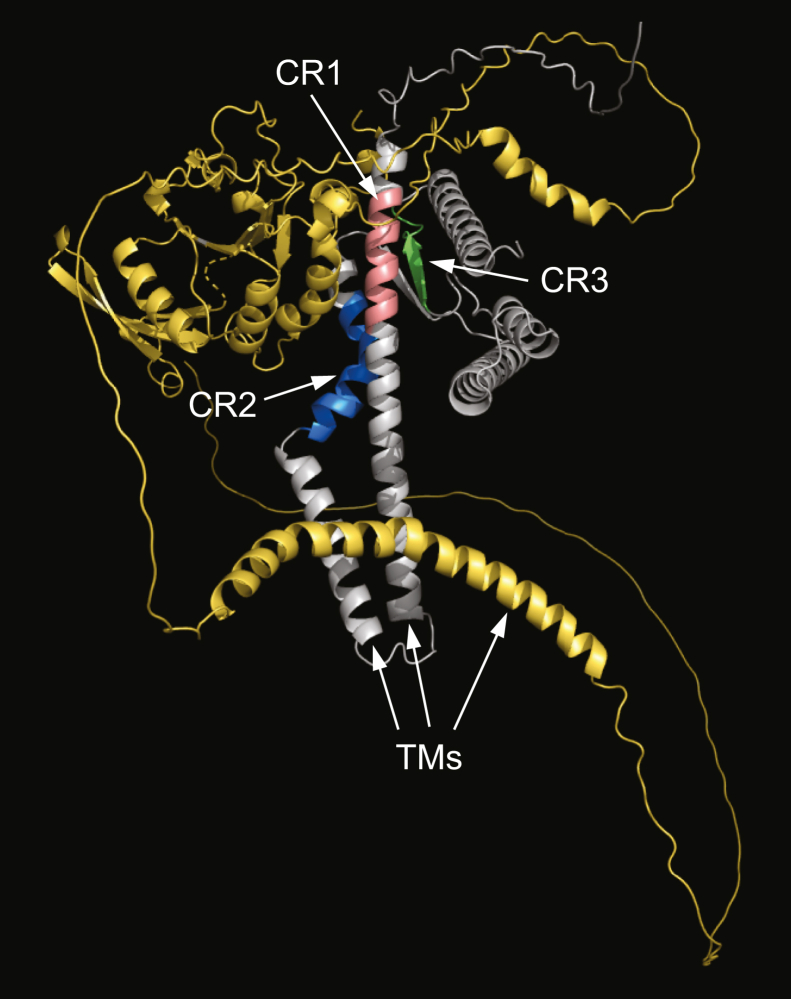
**Predicted structure of the Nem1–Spo7 complex.** The predicted AlphaFold structure of the Nem1 (UniProt: P38757)–Spo7 (UniProt: P18410) complex was generated by UCSF Chimera molecular modeling system and visualized by the PyMol program. The Nem1 and Spo7 structures are shown in *yellow* and *gray*, respectively. The positions of CR1 (*pink*), CR2 (*blue*), and CR3 (*green*) in Spo7 as well as the transmembrane regions (TMs) in Nem1 and Spo7 are indicated.

Of course, understanding how Spo7 interacts with Nem1 to form the complex would be better understood if *bona fide* structures of both proteins were available. However, in the absence of their structures, we have utilized the UCSF Chimera (72) and AlphaFold (73, 74) algorithms to predict the Nem1 (UniProt: P38757)–Spo7 (UniProt: P18410) complex (Fig. 8). The model shows the predicted positions of the Spo7 conserved regions being in proximity to Nem1.

The work presented here advances our understanding of the importance of the conserved regions in Spo7 for its complex formation with Nem1. Previous work indicated that the C-terminal half of Nem1 was important for its interaction with Spo7 (30). Additional studies are needed to understand what residues within Nem1 are important for the complexation with Spo7. It is known that the acidic tail of Pah1 is important for its transient association with the Nem1–Spo7 complex (29), but it is unclear how the association is governed by Spo7. Current studies are directed to address these questions.

## Experimental procedures

### Reagents

All growth media were sourced from Difco Laboratories. Plasmid miniprep columns and gel extraction kits were from Qiagen. Carrier DNA for yeast transformation and DNA size markers were purchased from Clontech. Enzyme reagents for DNA manipulation and the Q5 site-directed mutagenesis kit were from New England Biolabs. Bio-Rad was the source of reagents required for Western blotting, Bradford protein assay reagent, and molecular mass protein standards. Polyvinylidene difluoride membrane, enhanced chemifluorescence substrate for Western blotting. and IgG-Sepharose beads were from GE Healthcare. Silica gel 60 TLC plates, ampicillin, bovine serum albumin, 2-mercaptoethanol, PCR primers for mutagenesis, nucleotides, Triton X-100, and protease inhibitors were sourced from MilliporeSigma. Thermo Fisher Scientific supplied alkaline phosphatase–conjugated goat anti-rabbit IgG antibody (product number: 31340; lot number: NJ178812) and BODIPY 493/503. Radiochemicals and Scintillation counting supplies were obtained from PerkinElmer Life Sciences and National Diagnostics, respectively. Rabbit anti-Spo7 antibody directed against the amino acid sequence EDDLRRQAHEQK (residues 58–69) or REGARRRKQAHELRPKSE (residues 242–259) (37), rabbit anti-Nem1 antibody directed against the sequence KESDQNQERKNSVPKKPKA (residues 65–83) or ERRVKHTDKRNRGSN (residues 127–141) (37), rabbit anti-Pah1 antibody directed against the sequence TSIDKEFKKLSVSKAGA (residues 778–794) (19), and rabbit anti-Cho1 (phosphatidylserine synthase) antibody directed against the sequence MVESDEDFAPQEFPH (residues 1–15) (75) were prepared by BioSynthesis, Inc. The IgG fraction of each antibody was isolated from the serum and used in this work. All other chemicals were reagent grade or better.

### Plasmids, strains, and DNA manipulations

The plasmids used in this study are listed in Table 1. Standard methods were used for the isolation of plasmid DNA and its manipulation (76, 77, 78). Transformation of *Escherichia coli* (77) and *S. cerevisiae* (79) with plasmid DNA was performed as described previously. Plasmid pGH443 (37), derived from pRS415 (80), directs the low-copy expression of *SPO7* from its own promoter in yeast. Plasmids YCplac111-*GAL1/10*-*NEM1-PtA* and pRS314-*GAL1/10*-*SPO7* were used for the galactose-induced overexpression of protein A-tagged Nem1 and Spo7, respectively. Derivatives of pGH443 and pRS314-*GAL1/10*-*SPO7* were constructed by site-directed mutagenesis with primers designed using the NEBaseChanger online software. All mutations were confirmed by DNA sequencing. Plasmid pGH452 bearing *PAH1*-PtA under the control of *GAL1* promoter was derived from a high-copy number *Escherichia coli*/yeast shuttle vector, pYES2 (81).

The strains used in this study are listed in Table 2. *E. coli* strain DH5α was used for plasmid amplification and maintenance. All *S. cerevisiae* strains were derived from RS453 (82). GHY67 (37) is a *spo7*Δ*::URA3* mutant that was used for the plasmid-directed expression of WT and mutant Spo7 proteins. The *pah1*Δ*::natMX4* disruption cassette, which was generated by PCR amplification from pAG25 (EUROSCARF) as described previously for the *app1*Δ*::natMX4* cassette (83), was transformed into the *nem1*Δ *spo7*Δ mutant (SS1010) (30) to construct the *nem1*Δ *spo7*Δ *pah1*Δ mutant (GHY85) by one-step gene replacement (84). The nourseothricin (100 μg/ml)-resistant transformant cells were analyzed by PCR to confirm the gene replacement. The triple mutant was used for the overexpression of the protein A-tagged Nem1–Spo7 complex. The *pah1*Δ mutation prevents the growth inhibition caused by the overexpression of the protein phosphatase complex (32). The *pah1*Δ *nem1*Δ mutant (SS1132) (19) was used for pGH452-mediated overexpression of the phosphorylated Pah1 and its purification (81). The *nem1*Δ mutation yields cells devoid of the Nem1–Spo7 complex, ensuring the hyperphosphorylation of Pah1 (32, 81).

**Table 2:** Strains used in this study

Strain	Relevant characteristics	Source or reference
*E. coli*		
DH5α	F^-^ φ80d*lacZ*ΔΜ15Δ (*lacZYA*-*argF*)U169 *deoR rec*A1 *end*A1 *hsd*R17(*r*_k_^−^*m*_*k*_^+^) *pho*A *sup*E44 λ^−^*thi*-1 *gyr*A96 *rel*A1	(77)
*S*. *cerevisiae*		
RS453	*MATa ade2-1 his3-11,15 leu2-3112 trp1-1 ura3-52*	(82)
Derivatives		
GHY67	*spo7*Δ*::URA3*	(37)
SS1010	*nem1::HIS3 spo7::HIS3*	(31)
GHY85	*nem1::HIS3 spo7::HIS3 pah1*Δ*::natMX4*	This study
SS1132	*pah1*Δ*::TRP1 nem1*Δ*::HIS3*	(19)

### Growth conditions

*E. coli* cells were grown at 37 °C in Luria–Bertani broth (1% tryptone, 0.5% yeast extract, 1% NaCl, pH 7.0) containing 100 μg/ml ampicillin to select transformants carrying plasmids. Bacterial and yeast growth in liquid medium was estimated spectrophotometrically by absorbance at 600 nm. Yeast cells were cultured using standard methods (76, 77); they were routinely grown at 30 °C in either yeast extract–peptone–dextrose (YPD) (1% yeast extract, 2% peptone, and 2% dextrose) or synthetic complete media. Cells carrying plasmids were selected for or maintained by growth in synthetic dropout media without leucine (SC-Leu). Unless indicated otherwise, media contained 2% dextrose as a carbon source. For temperature sensitivity assay, plasmid-carrying cells were serially diluted (10-fold) in SC-Leu media and spotted onto SC-Leu or YPD agar plates. Cell growth was scored after 3 days of incubation at 30 and 37 °C. The growth patterns on each medium were similar; the data presented were from the YPD plates. For the galactose-induced expressions of protein A-tagged Nem1 and Spo7 (WT and mutant forms), cells were grown to the exponential phase in SC-Leu-Trp medium with 2% dextrose, washed and resuspended in SC-Leu-Trp medium containing 2% galactose/1% raffinose, and incubated for 14 h.

### Lipid labeling and analysis

*S. cerevisiae* cells were labeled to steady state with [2-^14^C]acetate (85); lipids were extracted from stationary phase cells by the method of Bligh and Dyer (86) as described by Fakas *et al.* (87). Lipids were resolved by one-dimensional TLC on silica gel plates using the solvent system hexane/diethyl either/glacial acetic acid (40:10:1, v/v) (88). The resolved lipids were visualized by phosphorimaging with a Storm 860 Molecular Imager (GE Healthcare) and analyzed by ImageQuant software using a standard curve of [2-^14^C]acetate. The identities of radiolabeled TAG and total phospholipids were confirmed by comparison with the migration of authentic standards visualized by iodine vapor staining.

### Analysis of lipid droplets

*S. cerevisiae* cells were grown in SC-Leu media at 30 °C to the stationary phase and then incubated with 1 μg/ml BODIPY 493/503 for 30 min to visualize lipid droplets (40). The fluorescent signal from the lipid droplets was examined under a Nikon Eclipse Ni-U microscope using an EGFP/FITC/Cy2/AlexaFluor 488 filter and recorded by a DS-Qi2 camera. Image analysis was performed with the NIS-elements BR software. The number of lipid droplets was determined by examination from ≥4 fields of view (≥200 cells).

### Preparation of cell extracts, subcellular fractionation, and enzyme purification

All steps were performed at 4 °C. Yeast cultures were harvested by centrifugation at 1500*g* for 5 min. The collected cells were washed with water and resuspended in lysis buffer (50 mM Tris–HCl [pH 7.5], 10% glycerol, 10 mM 2-mercaptoethanol, 1 mM Na_2_EDTA, 0.5 mM phenylmethylsulfonyl fluoride, 1 mM benzamidine, 5 μg/ml aprotinin, 5 μg/ml leupeptin, and 5 μg/ml pepstatin). Glass beads (0.5 mm diameter) were added to the cell suspension, which was then subjected to five repeats of 1 min burst and 2 min cooling using a BioSpec Products Mini-Beadbeater-16 (89). The cell lysates were centrifuged at 1500*g* for 10 min to separate unbroken cells and cell debris (pellet) from cell extracts (supernatant). The cell extract was centrifuged at 100,000*g* for 1 h to separate the cytosol (supernatant) from the membrane (pellet). The membrane fraction, which was used for the Pah1 translocation assay, was resuspended in 50 mM Tris–HCl buffer (pH 7.5) containing 10 mM MgCl_2_, 10 mM 2-mercaptoethanol, 10% glycerol, and protease inhibitors.

Protein A-tagged Nem1–Spo7 complex was purified from the *nem1*Δ *spo7*Δ *pah1*Δ mutant (GHY85) expressing plasmids YCplac111-*GAL1/10*-*NEM1*-PtA and pRS314-*GAL 1/10-SPO7* (WT or mutant forms) by affinity chromatography with IgG-Sepharose as described by Siniossoglou *et al.* (90) with minor modifications (31). Phosphorylated Pah1 was purified from the *pah1*Δ *nem1*Δ mutant (SS1132) expressing plasmid pGH452 by IgG-Sepharose affinity chromatography, anion exchange chromatography, and size-exclusion chromatography (81). Purified enzyme preparations were stored at −80 °C.

### SDS-PAGE and immunoblot analysis

Standard procedures were used for SDS-PAGE (91) and immunoblotting with a polyvinylidene difluoride membrane (92, 93). The samples for immunoblotting were normalized to total protein loading. Protein transfer from polyacrylamide gels to polyvinylidene difluoride membranes was monitored by staining with Ponceau S. The blots were probed with rabbit anti-Nem1 (1 μg/ml), anti-Spo7 (1 μg/ml), anti-Pah1 (2 μg/ml), or anti-Cho1 (0.25 μg/ml) antibody, followed by goat anti-rabbit IgG antibody conjugated with alkaline phosphatase at the dilution of 1:5000. Immune complexes were detected with the enhanced chemifluorescence immunoblotting substrate. Fluorimaging with a Storm 865 Molecular Imager was used to visualize fluorescence signals from immunoblots. A standard curve ensured that the immunoblot signals were in the linear range of detection.

### Nem1–Spo7 protein phosphatase assay

The Nem1–Spo7 phosphatase activity of the membrane fraction prepared from GHY85 cells expressing protein A-tagged Nem1–Spo7 (WT or mutant forms) was assessed by the electrophoretic mobility of Pah1 upon SDS-PAGE using 6% polyacrylamide gels (40). The reaction mixture contained 100 mM sodium acetate (pH 5.0), 10 mM MgCl_2_, 1 mM DTT, 20 μg membranes, and 2.5 ng Pah1 in a total volume of 20 μl. Phosphorylated and dephosphorylated forms of Pah1 were visualized by immunoblotting with anti-Pah1 antibody.

### Protein determination

Protein amounts were estimated by the protein-dye binding assay using bovine serum albumin as the standard (94).

### Data analysis

The statistical analysis of data was determined with Microsoft Excel software. The *p* value <0.05 was taken as a significant difference.

## Data availability

All data are contained within the article.

## Conflict of interest

The authors declare that they have no conflicts of interest with the contents of this article.
